# Healthy Tissue Damage Following Cancer Ion Therapy: A Radiobiological Database Predicting Lymphocyte Chromosome Aberrations Based on the BIANCA Biophysical Model

**DOI:** 10.3390/ijms221910877

**Published:** 2021-10-08

**Authors:** Alessia Embriaco, Ricardo Ramos, Mario Carante, Alfredo Ferrari, Paola Sala, Valerio Vercesi, Francesca Ballarini

**Affiliations:** 1 ISezione di Pavia, INFN (Italian National Institute for Nuclear Physics), Via Bassi 6, 27100 Pavia, Italy; alessia.embriaco@pv.infn.it (A.E.); ricardoramos85@gmail.com (R.R.); mario.carante@unipv.it (M.C.); valerio.vercesi@pv.infn.it (V.V.); 2 Istituto Nazionale di Metrologia delle Radiazioni Ionizzanti, ENEA, 00123 Roma, Italy; 3 Physics Department, University of Pavia, Via Bassi 6, 27100 Pavia, Italy; 4 University Hospital Heidelberg, 69120 Heidelberg, Germany; alfredo.ferrari@mi.infn.it; 5 Gangneung-Wonju National University, Gangneung 25457, Korea; 6 Sezione di Milano, INFN (Italian National Institute for Nuclear Physics), Via Celoria 16, 20133 Milano, Italy; paola.sala@mi.infn.it

**Keywords:** cancer, hadrontherapy, healthy tissue damage, chromosome aberrations, peripheral blood lymphocytes, biomarkers, cell death, relative biological effectiveness, Monte Carlo, biophysical modelling

## Abstract

Chromosome aberrations are widely considered among the best biomarkers of radiation health risk due to their relationship with late cancer incidence. In particular, aberrations in peripheral blood lymphocytes (PBL) can be regarded as indicators of hematologic toxicity, which is a major limiting factor of radiotherapy total dose. In this framework, a radiobiological database describing the induction of PBL dicentrics as a function of ion type and energy was developed by means of the BIANCA (BIophysical ANalysis of Cell death and chromosome Aberrations) biophysical model, which has been previously applied to predict the effectiveness of therapeutic-like ion beams at killing tumour cells. This database was then read by the FLUKA Monte Carlo transport code, thus allowing us to calculate the Relative Biological Effectiveness (RBE) for dicentric induction along therapeutic C-ion beams. A comparison with previous results showed that, while in the higher-dose regions (e.g., the Spread-Out Bragg Peak, SOBP), the RBE for dicentrics was lower than that for cell survival. In the lower-dose regions (e.g., the fragmentation tail), the opposite trend was observed. This work suggests that, at least for some irradiation scenarios, calculating the biological effectiveness of a hadrontherapy beam solely based on the RBE for cell survival may lead to an underestimation of the risk of (late) damage to healthy tissues. More generally, following this work, BIANCA has gained the capability of providing RBE predictions not only for cell killing, but also for healthy tissue damage.

## 1. Introduction

Exposing living cells to ionizing radiation can induce chromosome aberrations (CAs), consisting of large-scale genome rearrangements following incorrect rejoining (“mis-rejoining”), or un-rejoining, of chromosome fragments created by energy depositions in the DNA. In particular, an increase in the CA yield in peripheral blood lymphocytes (PBL) is considered as an indicator of hematologic toxicity [1] since lymphocytes circulate in the blood vessels and are distributed throughout the body in the hematopoietic tissue components [2].

Hematopoietic tissue damage is a major limiting factor of radiotherapy total dose both for acute morbidity and for secondary cancer risk [3,4] since CAs are well-correlated to late cancer incidences [5,6,7,8,9,10]. In particular, Durante et al. [1] found a reduced level of PBL aberrations in patients treated with C-ions with respect to those treated with X-rays, suggesting a reduced risk of bone marrow morbidity. Subsequently, the same research group found that the frequency of aberrant lymphocytes in C-ion-treated lung cancer patients [11] and prostate cancer patients treated by C-ions and/or IMRT [12] was correlated to the size of the irradiation field.

To evaluate healthy tissue damage following ion therapy by means of PBL aberrations, in this work we applied the BIANCA (BIophysical ANalysis of Cell death and chromosome Aberrations) biophysical model to develop a radiobiological database describing the induction of lymphocyte chromosome aberrations as a function of ion type and energy, as well as photons as a reference. This database, which in principle can be read by any radiation transport code or Treatment Planning System (TPS), was then read by the FLUKA Monte Carlo code [13,14,15,16,17,18], allowing us to predict the RBE for chromosome aberration induction in each voxel of therapeutic-like C-ion beams and to compare such predictions with calculations of the RBE for tumour cell survival performed in a previous work [19].

## 2. Results and Discussion

### 2.1. Benchmark with Experimental Data and Development of the Radiobiological Database

**Figure 1:**
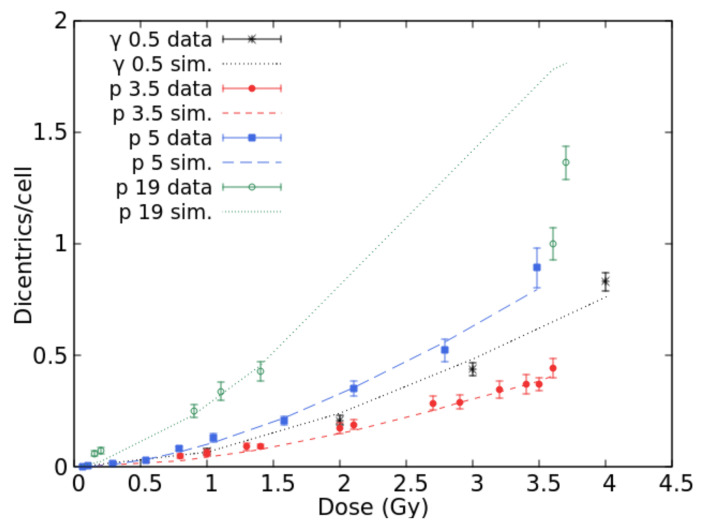
Mean number of dicentrics per cell versus dose for gamma-rays and protons (3.5, 5.0, and 19.0 keV/μm). The points with error bars are experimental data, the lines are simulation outcomes.

Many papers are available in the literature on chromosome aberration induction in human lymphocytes cultured in vitro. In particular, Bauchinger and Schmid [20] reviewed several published data concerning dicentric induction by various radiation qualities in the LET range 0.5–150 keV/μm. Figure 1 shows the comparisons between BIANCA simulations and the data reviewed in [20] for Cs-137 gamma-rays, published in [21], 3.5 keV/μm protons [22] and 19.0 keV/μm protons [23], as well as the 5.0 keV/μm proton data reported in [24]. The 5.3 keV/μm proton data published in [23] were also considered but not displayed, to avoid making the figure too difficult to read. A good agreement with the data was found for gamma rays, which in the following were used as a reference for RBE calculation. Good agreement was also found for 3.5 keV/μm protons and 5.0 keV/μm protons, for which the simulation outcomes are within the experimental error bars in most cases. For 19 keV/μm protons, the simulated dicentric yields at the higher doses are higher than the observed ones. Although the underlying reasons are still unclear, it is worth mentioning that the present work focuses on doses not exceeding 3 Gy to stay within the dose range of interest for a typical hadrontherapy fraction. The figure also shows that 3.5 keV/μm protons were less effective than gamma rays, despite the lower LET of the latter (0.5 keV/μm according to [20]). This may be explained by considering that these data have been published in different papers, possibly implying differences in the cells’ radiosensitivity and/or in the experimental conditions.

**Figure 2:**
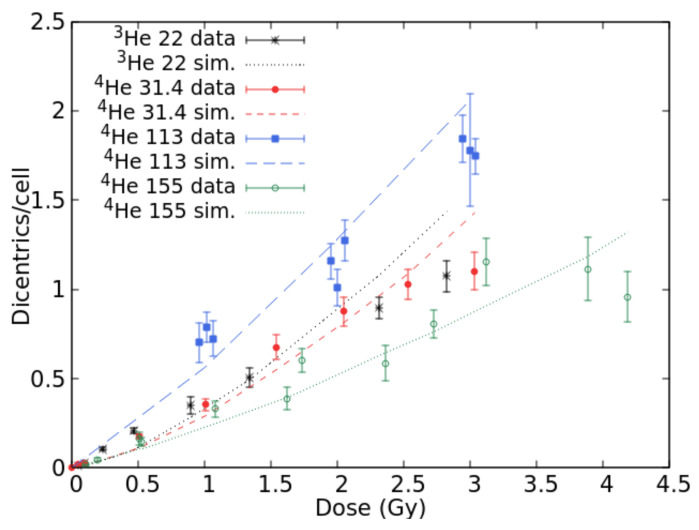
Mean number of dicentrics per cell versus dose for He-ion beams with LET values of 22.0, 31.4, 113.0, and 155.0 keV/μm. The points with error bars are experimental data, and the lines are simulation outcomes.

Data on lymphocyte dicentrics by He-ions were found in [24] (for 22.0 keV/μm ^3^He ions), [25] (24.0 keV/μm ^3^He ions and 140.0 keV/μm alpha particles), [26] (31.4 keV/μm ^4^He ions), [27] (113.0 keV/μm alpha particles), [28] (150.0 keV/μm alpha particles), and [29] (155.0 keV/μm alpha particles). Figure 2 reports simulation outcomes and experimental data at 22.0, 31.4, 113.0 and 155.0 keV/μm. Simulations were also carried out at 24.0, 140.0 and 150.0 keV/μm, although the results were not displayed to avoid making the figure too “crowded”; furthermore, at 24.0 and 140.0 keV/μm, the raw data were not available because the authors only reported the data fits. At 22.0 keV/μm, the simulations showed a tendency to underestimate the data at lower doses and overestimate those at higher doses, suggesting a saturation that might be due to a competition between dicentrics and complex exchanges. However, the agreement in the dose range 1–1.5 Gy is acceptable. For 31.4 keV/μm He-ions, the simulations reproduced the data quite well up to 2.5 Gy, whereas at 3 Gy, the simulated dicentric yield was higher than the observed one. Again, the data suggest a saturation-like pattern that is not reproduced by the simulations. Indeed, the data at 22.0 and 31.4 keV/μm are not very different, although one would expect a higher effectiveness at 31.4 keV/μm. Possible explanations may include the fact that these data have been published in different papers. The data at 113.0 keV/μm were well-reproduced by the simulations, and a good agreement with the data was also found at 155.0 keV/μm where the simulations are within the error bars with few exceptions. The fact that the experimental yields at 155.0 keV/μm are lower than those at lower LET may be explained by considering that these data have not been obtained by Premature Chromosome Condensation (PCC), and thus are likely to be influenced by mitotic delay and/or interphase death.

**Figure 3:**
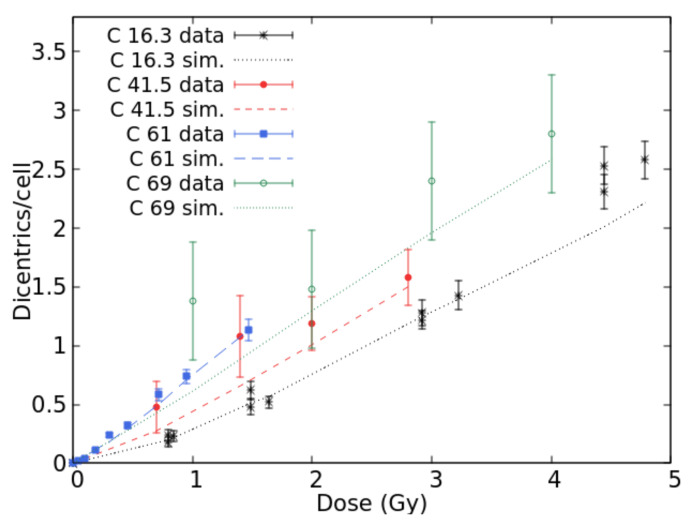
Mean number of dicentrics per cell versus dose for C-ion beams with LET values of 16.3, 41.5, 61.0, and 69.0 keV/μm. The points with error bars are experimental data, and the lines are simulation outcomes.

Concerning lymphocyte dicentrics by C-ions, experimental data were found in [30] (LET = 16.3 keV/μm), [31] (22.4, 41.5 and 69.9 keV/μm), [32] (34.6 keV/μm), and [26] (61.0 keV/μm). Figure 3 shows the comparison between BIANCA simulations and the data at 16.3, 41.5, 61.0, and 69.9 keV/μm. The results at 22.0 and 34.6 keV/μm were not displayed to avoid making the figure too difficult to read. The simulations were in good agreement with the data for each considered LET value since they were within the error bars for most points. Again, the fact that the data at 69.9 keV/μm do not seem higher than those at 61.0 keV/μm despite the higher LET may be explained by considering that the two data sets have been obtained by different laboratories. Furthermore, the large error bars associated with the 69.9 keV/μm data make the interpretation more difficult.

**Figure 4:**
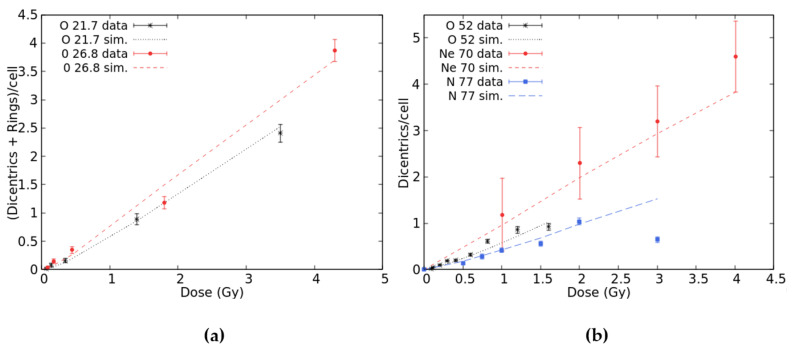
Mean number of dicentrics plus rings per cell versus dose-induced by 21.7 and 26.8 keV/μm O-ions (panel **a**) and mean number of dicentrics per cell versus dose-induced by 52 keV/μm O-ions, 70 keV/μm Ne-ions, and 77 keV/μm N-ions (panel **b**). The points with error bars are experimental data, and the lines are simulation outcomes.

Oxygen data were found in [25] (LET = 49.0 keV/μm), [26] (52.0 keV/μm) and [33] (21.7 and 26.9 keV/μm). Furthermore, Govorun et al. [34] irradiated human lymphocytes with 77 keV/μm N-ions, whereas Ohara et al. [31] used 70 keV/μm Ne-ions. Figure 4a reports the results for 21.7 and 26.9 keV/μm O-ions for which dicentrics have been scored together with rings; in both cases, good agreement was found between the simulations and data. Figure 4b reports the results for 52 keV/μm O-ions, 70 keV/μm Ne-ions, and 77 keV/μm N-ions. The results for 49 keV/μm O-ions were not displayed because they would not add information with respect to 52 keV/μm O-ions since the two LET values are very similar. Furthermore, the raw data were not available because only the data fit was published. For 52 keV/μm oxygen and neon, there was a good agreement between simulations and data in the whole considered dose range. Concerning nitrogen, the simulations reproduced the data up to 2 Gy, whereas at 3 Gy, the BIANCA outcome is much higher than the experimental point. However, this point is much lower with respect to the trend shown by the points at lower doses. The fact that the experimental dicentric yields for 77 keV/μm N-ions are lower than those for 52 keV/μm O-ions and 70 keV/μm Ne-ions is not easy to interpret. Overall, considering that the goal of the present work was to focus on doses up to 3 Gy, the results reported in Figure 1, Figure 2, Figure 3 and Figure 4 show that BIANCA is capable of reproducing the data for all considered particle types and LET values.

**Figure 5:**
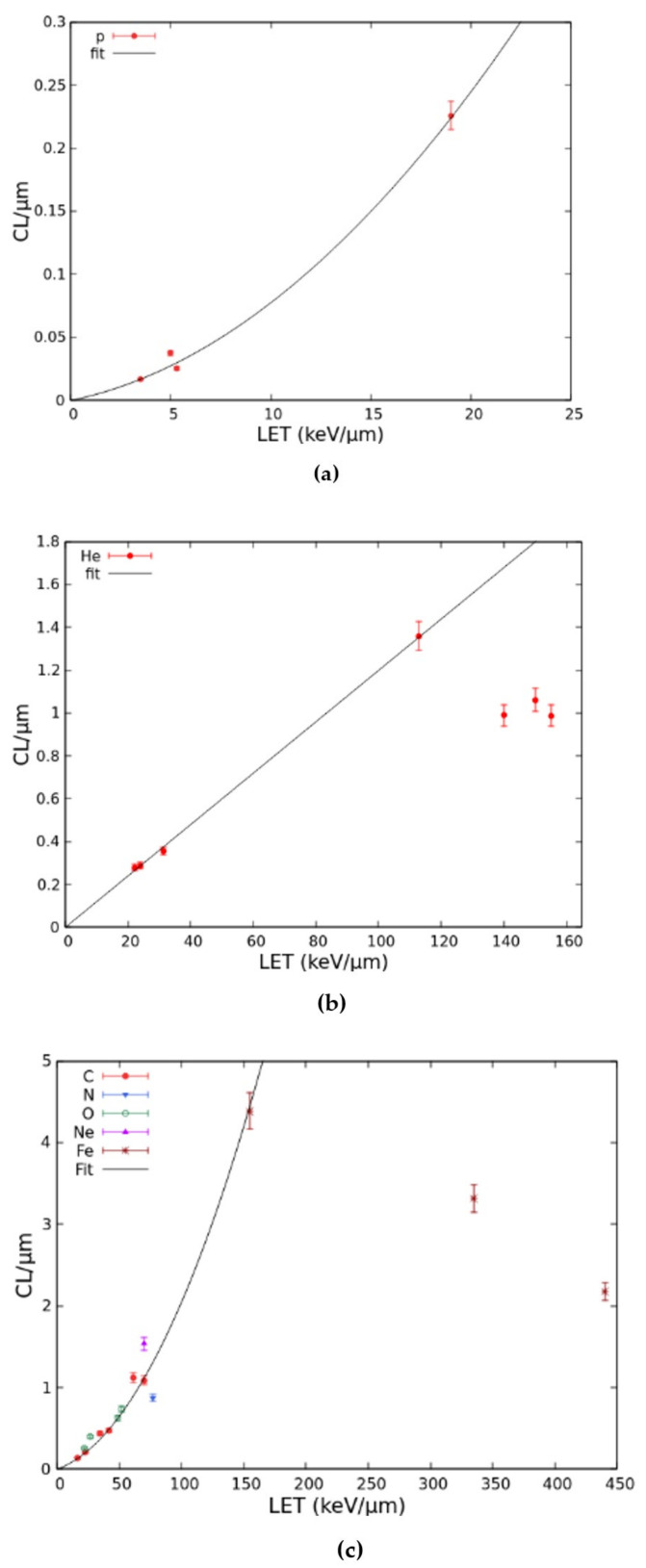
LET-dependence of the CL yield for protons (**a**), He-ions (**b**), and heavy ions (**c**). Each point represents a CL yield, and the lines are fits as described in the text.

The dose–response curves described above were obtained by separately adjusting the CL parameter for each ion type and each LET value, following comparisons with the data. The LET-dependence of these CL yields (mean number of CLs per micrometre) was then analysed and fitted for protons, He-ions, and heavy ions, where “heavy” refers to all ions with atomic numbers equal or higher than 6. These CL yields, together with the corresponding fits, are reported in Figure 5. As shown by panel (a), the proton CL yield was fitted by a linear-quadratic function in the considered LET range. For He-ions (panel (b)), the CL yields found above 113 keV/μm, which were lower than the yield at 113 keV/μm, were excluded from the fit to avoid underestimating the damage at high LET; the remaining yields were fitted by a linear function. Finally, the CL yields for heavy ions (panel (c)) were fitted by a linear-quadratic function up to 155 keV/μm, after excluding from the fit the points above 155 keV/μm, analogous to what was carried out for He-ions.

This fitting procedure provided the CL yields to predict dicentric dose–response curves for monochromatic beams of protons, He-ions, or heavy ions at many different LET values without performing any further parameter adjustment. Each of these (simulated) curves was then fit by Equation (2) and the linear and quadratic coefficients deriving from such fit were stored in a table that constitutes a radiobiological database describing lymphocyte dicentric induction by different monochromatic ion beams.

### 2.2. RBE Evaluation for Therapeutic-Like Carbon Beams

**Figure 6:**
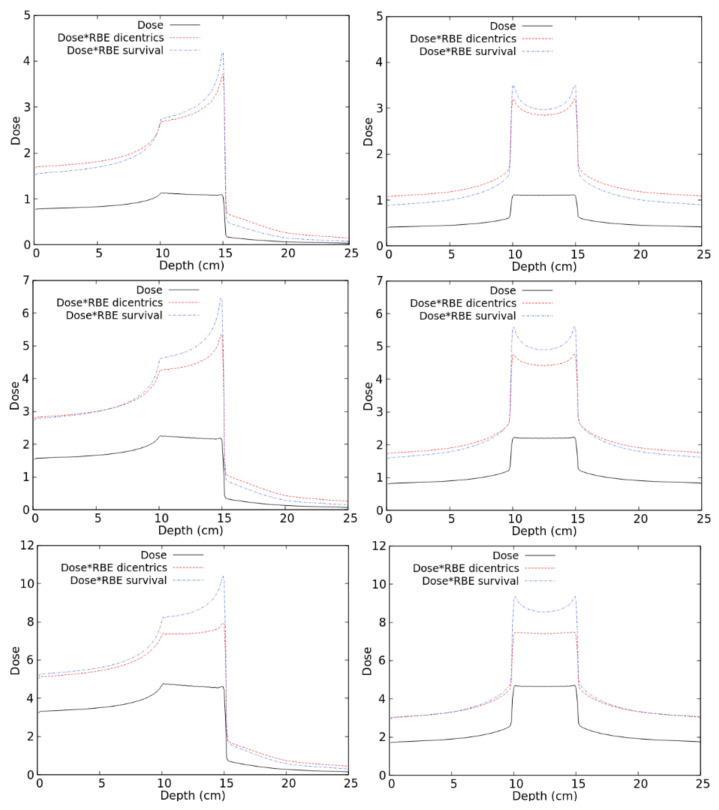
Distribution of absorbed doses and RBE-weighted doses (both for lymphocyte dicentrics and for cell death) for different C-ion fields as described in the text.

To predict the RBE for lymphocyte dicentrics along therapeutic-like carbon beams, distributions of the absorbed dose in water were calculated by FLUKA, and the RBE for dicentrics in each irradiated voxel was calculated by reading the dicentric radiobiological database described above. As an example, Figure 6 shows the obtained distributions of RBE-weighted dose, as well as the corresponding distributions of absorbed dose for a uniform absorbed dose of 1, 2, or 4 Gy in a 5 cm SOBP. The irradiation was performed either with a mono-directional beam (left panels) or by two opposing beams (right panels). In addition to the RBE-weighted dose for dicentrics calculated in this work, each panel also reports the RBE-weighted dose for chordoma cell survival obtained in a previous study [19].

As a general trend, in the regions receiving higher doses (such as the SOBP and, for the 4-Gy case, the entrance channel), the RBE-weighted dose for dicentrics was lower than that for cell survival. The opposite behaviour was found in the low-dose regions, such as the fragmentation tails where the dicentric RBE was higher than the cell survival RBE. This may be explained by considering that the photon dose–response for lymphocyte dicentrics tends to show a lower linear coefficient (and sometimes a higher quadratic coefficient, too), and thus a lower alpha/beta ratio with respect to cell survival curves. As a consequence, lymphocyte dicentrics tend to show a higher RBE at low doses and a lower RBE at higher doses. In principle, another possible reason for the observed difference between dicentric RBE and cell survival RBE may be related to differences in the reference photons. While the dicentrics of the RBE were calculated based on ^137^Cs gamma-rays, high-energy (6 MV) X-rays have been used for the chordoma cell survival study presented in [19] and was considered in the present work for comparison. However, this is expected to have a minor impact because the possible differences due to the considered photon type (gamma-rays or high-energy X-rays) are less important than the differences between the considered endpoint (lymphocyte dicentrics or cell survival). Indeed, the alpha coefficient for photon-induced lymphocyte dicentrics is typically in the order of 10^−2^ Gy^−1^ regardless of the photon energy. On the contrary, the alpha coefficient for most tumour cell survival data, including the chordoma data considered by us, in which we used α_X_ = 0.159, is in the order of 10^−1^ Gy^−1^.

The results shown in Figure 6 suggest that, at least for some irradiation scenarios, calculating the RBE solely based on cell killing may underestimate the damage to healthy tissues. This seems to be particularly evident at lower doses and for mono-directional irradiations, whereas it tends to become less important at higher doses and/or in case of irradiation with two opposing beams. The latter occurs because, with two opposing beams, the dose in the healthy tissue regions derives from the contribution of the entrance channel of one beam plus the fragmentation tail of the other one.

## 3. Materials and Methods

### 3.1. General Aspects of the Model

BIANCA is a biophysical model, implemented as a Monte Carlo code, which simulates the induction of chromosome aberrations and cell death by different monochromatic ion beams, as well as photons [35,36]. The model is based on the following assumptions: (i) ionizing radiation can induce DNA “critical lesions” (CLs), where a CL is defined as a lesion that interrupts the chromatin fibre producing two (main) independent chromosome fragments; (ii) distance-dependent mis-rejoining of such fragments, or fragment un-rejoining, produces chromosomal aberrations; (iii) certain aberration categories (dicentrics, rings and large deletions, where “large” means visible when chromatin is condensed) lead to clonogenic cell death. A detailed discussion on these assumptions can be found in previous works [36].

Since the features of the DNA lesions leading to chromosomal aberrations and cell death are still unclear [37,38], in BIANCA the concept of CL is not defined a priori, and the CL yield (mean number of CLs per unit dose and unit DNA mass) is an adjustable parameter. As in our previous works on chromosome aberrations [39,40], in this work, the dependence of the chromosome-fragment rejoining probability, P, on the fragment initial distance, r, was also described by an exponentially-decreasing function of the form

P(r) = exp(−r/r_0_) (1)

where r_0_ depends on the considered cell type. Based on previous results on human lymphocytes [39], r_0_ was fixed to 0.8 μm. Further details, including the way to account for interphase nuclear architecture [41,42], can be found in [39,40,43,44,45].

### 3.2. Reproduction of Experimental Dose–Response Curves for PBL Dicentrics

As a first step of the work, we searched the literature for in vitro data on PBL chromosome aberrations induced by different (monochromatic) ion beams, as well as photons as a reference. Since most data deal with dicentrics, the attention was focused on this aberration category. Data on the following ion types were found: protons (in the LET interval between 3.5 and 19 keV/μm); He-ions (22–155 keV/μm); C-ions (16–70 keV/μm); N-ions (77 keV/μm); O-ions (22-52 keV/μm); Ne-ions (70 keV/μm); Fe-ions (155–440 keV/μm), which were included in view of future space radiation studies. Each experimental dose–response curve was reproduced by separately adjusting the CL parameter. Afterwards, the LET-dependence of the CL yield (mean number of CLs per unit ion path length, expressed as mean number of CLs per micrometre) was separately fitted for protons, He-ions, and heavy ions (C, N, O, Ne, Fe). For each ion, this fitting procedure provided the CL yields to predict dicentric dose–response at any LET value.

A large number of dicentric dose–response curves in the dose range 0–3 Gy was thus simulated for protons, He-ions, and heavy ions at many different LET values, within the following LET intervals: 2.5–30 keV/μm for protons, 5–110 keV/μm for He-ions, and 5–150 keV/μm for heavy ions. Each of these (simulated) curves was then fitted by the following linear-quadratic function:

Y(D) = αD + βD^2^ (2)

where Y(D) is the mean number of dicentrics per cell, D is the absorbed dose, and α and β are fitting coefficients. This expression is considered as a good description for dicentric dose–response at low and intermediate doses, which is the case of the present work, where the maximum dose was 3 Gy because higher doses are not of interest for healthy tissue damage following a single hadrontherapy fraction.

The linear and quadratic coefficients reported in Equation (2) were stored in a table as a function of ion type and LET, together with the two coefficients describing the photon dose–response (α = 0.020 Gy^−1^ and β = 0.047 Gy^−2^, obtained by reproducing the Cs-137 gamma-ray data reported in [20] and shown in Figure 1). Li-, Be-, and B-ions were also included in the database: at a given LET, the values of α and β for lithium were set equal to those for helium while the values for beryllium and boron were set equal to those for heavy ions. This approximation was due to the lack of experimental data on dicentric induction by these ion types; therefore, it was not possible to perform ad hoc simulations.

For each of the three considered ion categories (protons, He-ions, or heavy ions), the behaviour at higher LET values was described by the last available pair of α and β coefficients. For instance, the coefficients found for 110 keV/μm He-ions were also assigned to all He-ions with LET higher than 110 keV/μm, and those found for 150 keV/μm heavy ions were also assigned to all heavy ions with LET higher than 150 keV/μm. Although from a mechanistic point of view, this may overestimate the damage at very high LET, this choice was done to maintain a “conservative” approach. Since the main goal of the work consisted of evaluating the damage in healthy tissues, at very high LET, where no experimental data were available, we preferred to possibly overestimate such damage than to underestimate it.

### 3.3. Interface with the FLUKA Code

The table containing the dicentric linear and quadratic coefficients described above was then read by FLUKA (version 2020.0, www.fluka.org) (Accessed on 31 July 2020), exploiting a pre-existing interface between BIANCA and FLUKA. Specifically, according to FLUKA, whenever a certain radiation dose, D_i_, was deposited in a voxel (voxel size: 1 mm in the beam direction, 0.5 mm in the radial direction) by the i-th particle (i.e., a given particle type of given energy and thus given LET), FLUKA read the corresponding coefficients (α_i_ and β_i_) from the table and used them to calculate the average coefficients, (α and β) describing dicentric induction by the mixed field in that voxel based on the Theory of Dual Radiation Action [46] as described in [47], i.e.:

(3) $$\alpha =\frac{\sum_{i=1}^{n}\alpha_{i}D_{i}}{\sum_{i=1}^{n}D_{i}}$$

(4) $$\sqrt{\beta }=\frac{\sum_{i=1}^{n}\sqrt{\beta_{i}}D_{i}}{\sum_{i=1}^{n}D_{i}}$$

In each irradiated voxel, D_i_ is the absorbed dose due to the i-th particle calculated by FLUKA, α_i_ and β_i_ are the corresponding radiobiological coefficients provided by BIANCA, and α and β are their average values, which take into account that a mixed field is present in the voxel.

The RBE for dicentric induction in each voxel was then calculated as D_X_/D, where D is the total absorbed dose in the voxel and D_X_ was calculated as follows:

(5) $$D_{X}=\left[-\alpha_{X}+\sqrt{\alpha_{X}^{2}+4\beta_{X}Y}\right]/2\beta_{X}$$

In Equation (5), α_X_ and β_X_ are the coefficients describing dicentric induction by photons according to BIANCA, and *Y* is the (total) dicentric yield in the voxel.

## 4. Conclusions

In the framework of the evaluation of healthy tissue damage following hadrontherapy, a radiobiological database describing the induction of lymphocyte dicentrics as a function of ion type and energy, as well as dose, was developed by the BIANCA biophysical model, and the RBE for lymphocyte dicentric induction along therapeutic-like carbon beams was calculated following interface with the FLUKA code. A comparison with previous results showed that, while in the higher-dose regions (e.g., the SOBP) the RBE for lymphocyte dicentrics was lower than that for cell survival, in the lower-dose regions (e.g., the fragmentation tail), the dicentric RBE was higher than the cell-survival RBE. This suggests that, at least for some irradiation scenarios, calculating the beam biological effectiveness solely based on the RBE for cell survival may lead to an underestimation of healthy tissue damage. More generally, following this work, BIANCA has gained the capability of predicting RBE distributions not only for (tumour) cell killing but also for lymphocyte dicentrics, which in turn are related to (late) healthy tissue damage.

## Data Availability

Not applicable.
